# Cardiac hydatidosis presenting as complete atrioventricular block: multimodality imaging diagnosis despite conflicting serology—a case report

**DOI:** 10.1093/ehjcr/ytag789

**Published:** 2026-10-26

**Authors:** Diego Benjamín López Fazlic, Alejandro Quijada Fumero, Javier Orlando Quintero Ardila, Ana Laynez Carnicero, Luca Vannini

**Affiliations:** Department of Cardiology, Hospital Universitario Nuestra Señora de Candelaria, Carretera del Rosario 145, Santa Cruz de Tenerife 38010, Spain; Department of Cardiology, Hospital Universitario Nuestra Señora de Candelaria, Carretera del Rosario 145, Santa Cruz de Tenerife 38010, Spain; Department of Cardiology, Hospital Universitario Nuestra Señora de Candelaria, Carretera del Rosario 145, Santa Cruz de Tenerife 38010, Spain; Department of Cardiology, Hospital Universitario Nuestra Señora de Candelaria, Carretera del Rosario 145, Santa Cruz de Tenerife 38010, Spain; Department of Cardiology, Hospital Universitario Nuestra Señora de Candelaria, Carretera del Rosario 145, Santa Cruz de Tenerife 38010, Spain

**Keywords:** Cardiac hydatidosis, Complete atrioventricular block, Multimodal imaging, Case report

## Abstract

**Background:**

Cardiac echinococcosis accounts for fewer than 2% of *Echinococcus granulosus* cases but can cause life-threatening conduction disturbances. Serological sensitivity in cardiac involvement may fall to around 50% and may be even lower in calcified lesions, making multimodal imaging the diagnostic cornerstone.

**Case summary:**

A 16-year-old man from Senegal presented shortly after arrival by sea with complete atrioventricular (AV) block and general deterioration. Echocardiography identified a cystic lesion with hyperechoic border and anechoic interior in the basal interventricular septum. Cardiac magnetic resonance demonstrated STIR hyperintensity and peripheral late gadolinium enhancement (LGE). Computed tomography (CT) confirmed a 3 × 1.6 cm calcified annular cyst in the septum, a second lesion (1.7 × 1.2 cm) in the AV pericardial recess, with no other extracardiac locations identified. Serological results were conflicting: *E. granulosus* serology was negative, while *Entamoeba histolytica* serology was positive (1:1280), although stool examination was negative, suggesting prior exposure rather than active infection. The calcified cystic morphology supported probable hydatid disease as the primary diagnosis. Antiparasitic treatment was administered and a permanent dual-chamber pacemaker was implanted.

**Discussion:**

Multimodal imaging must take precedence over serology when results are absent or conflicting. The highly suggestive imaging pattern, calcified annular cyst, STIR hyperintensity, and peripheral LGE should not be dismissed in the presence of a misleadingly positive *Entamoeba* titre. With increasing migration from endemic regions to Europe, this diagnostic approach is of growing relevance for cardiologists.

Learning points

- In young immigrants from endemic regions, cardiac hydatidosis must be considered as a cause of complete atrioventricular block.
- Negative *Echinococcus* serology does not exclude cardiac hydatidosis; multimodal imaging remains essential for diagnosis despite conflicting serological results.

## Introduction

Cystic echinococcosis, caused by the larval stage of *Echinococcus granulosus*, is endemic across sub-Saharan Africa, the Middle East, and Southern Europe. Cardiac involvement occurs in fewer than 2% of all cases but can manifest with life-threatening complications including conduction disorders.^1,2^ Diagnosis is challenging: while serology reaches 80%–90% sensitivity for hepatic cysts, it may fall to around 50% in cardiac involvement, likely because an intact pericyst limits antigen release, and may be even lower in calcified lesions.^3,4^ We report a case of probable cardiac hydatidosis presenting as complete atrioventricular (AV) block in a 16-year-old immigrant from a highly endemic region, in which multimodal cardiovascular imaging established the diagnosis despite negative *E. granulosus* serology.

## Summary figure

**Figure:**
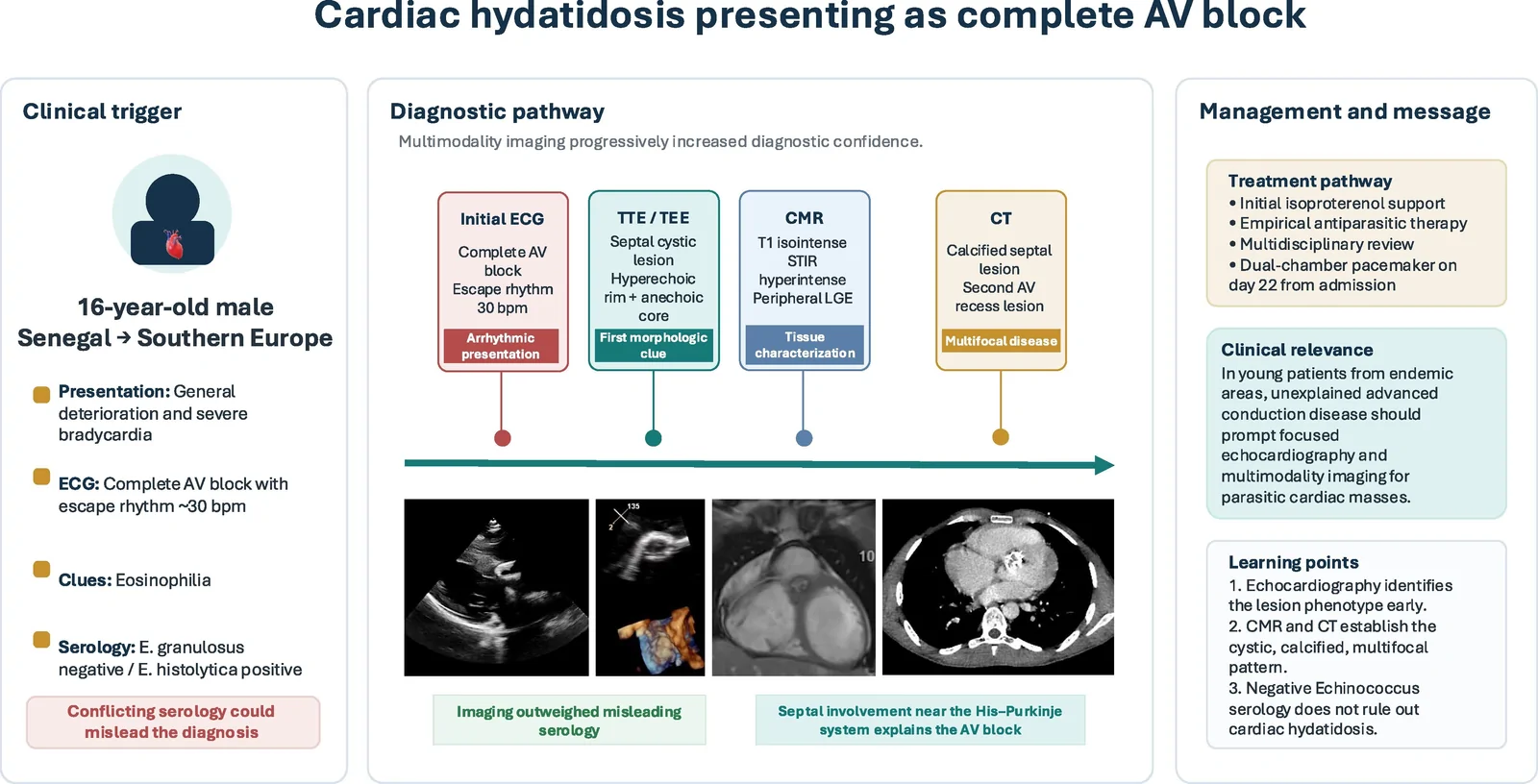


## Case presentation

A 16-year-old man from Senegal, recently arrived by sea, was transferred to our tertiary centre due to general deterioration and bradycardia. He reported an unknown cardiac condition diagnosed in his country of origin. The 12-lead electrocardiogram (*Figure 1*) demonstrated complete AV block with a ventricular escape rhythm at 30 b.p.m., requiring immediate intravenous isoproterenol. Physical examination revealed regular heart sounds and an aortic systolic murmur without signs of heart failure. Blood pressure was 132/57 mmHg and oxygen saturation 97%.

**Figure 1:**
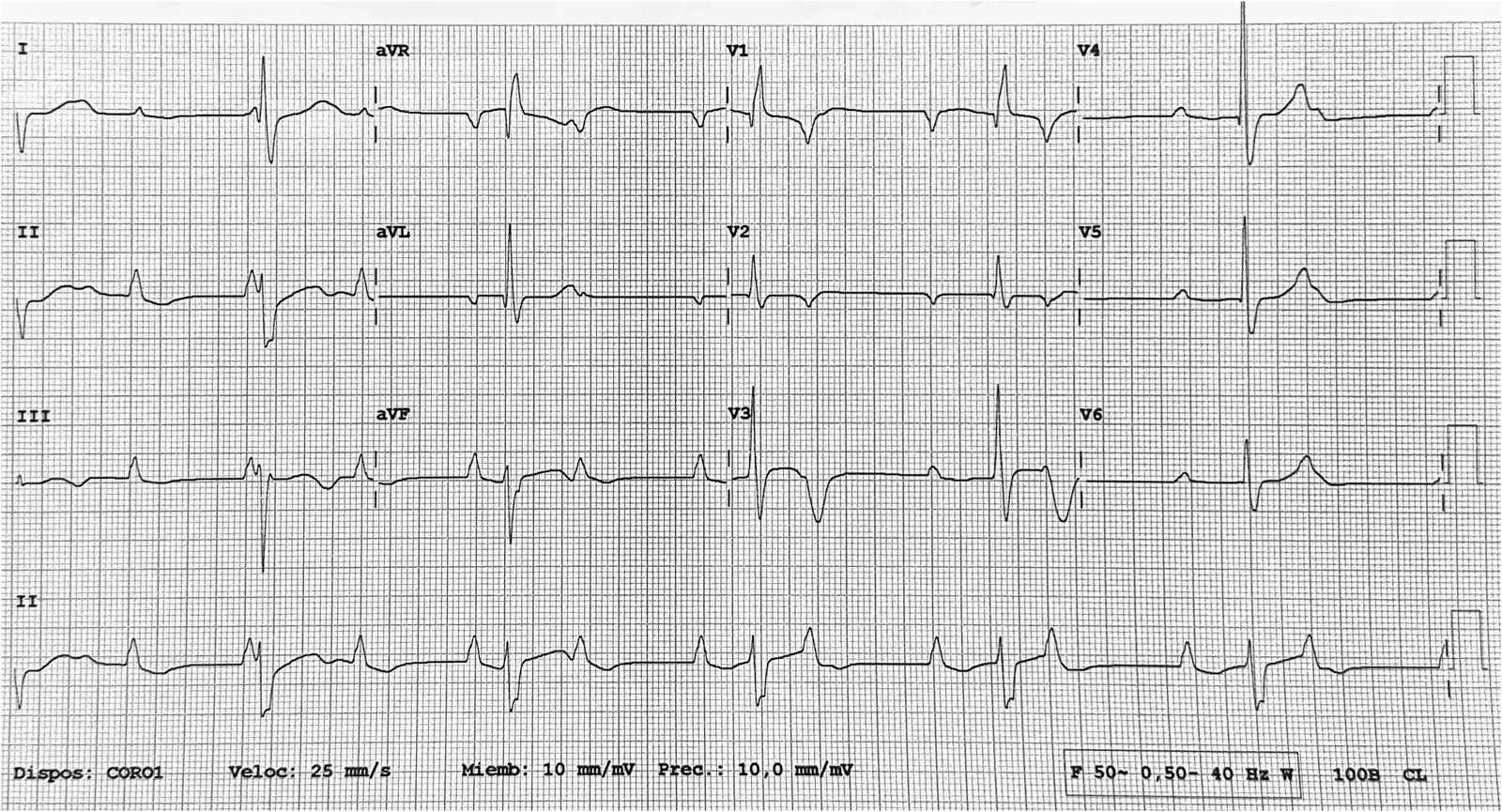
Twelve-lead electrocardiogram demonstrating complete atrioventricular block with a ventricular escape rhythm at 30 b.p.m.

Laboratory investigations showed mild elevation of C-reactive protein (2.42 mg/dL) and peripheral eosinophilia of 9%. Renal function and serum electrolytes were within normal limits. Serological testing for *E. granulosus* was performed by enzyme-linked immunosorbent assay and was negative. A repeat serological test was also negative. Extended infectious serology revealed a positive *Entamoeba histolytica* antibody titre (1:1280). However, PCR testing of stool samples was negative, suggesting prior exposure rather than active intestinal infection.

Transthoracic echocardiography revealed a mildly dilated left ventricle with preserved systolic function, septal-predominant hypertrophy, and a rounded lesion located in the basal anterior interventricular septum (*Figure 2*; Supplementary material online, *Video S1*). The lesion exhibited a well-defined hyperechoic border and a homogeneous anechoic interior, consistent with a cystic structure. No significant valvular disease was identified. Transoesophageal echocardiography confirmed these findings with enhanced anatomical resolution, confirming the cystic nature of the septal lesion (see Supplementary material online, *Video S2*).

**Figure 2:**
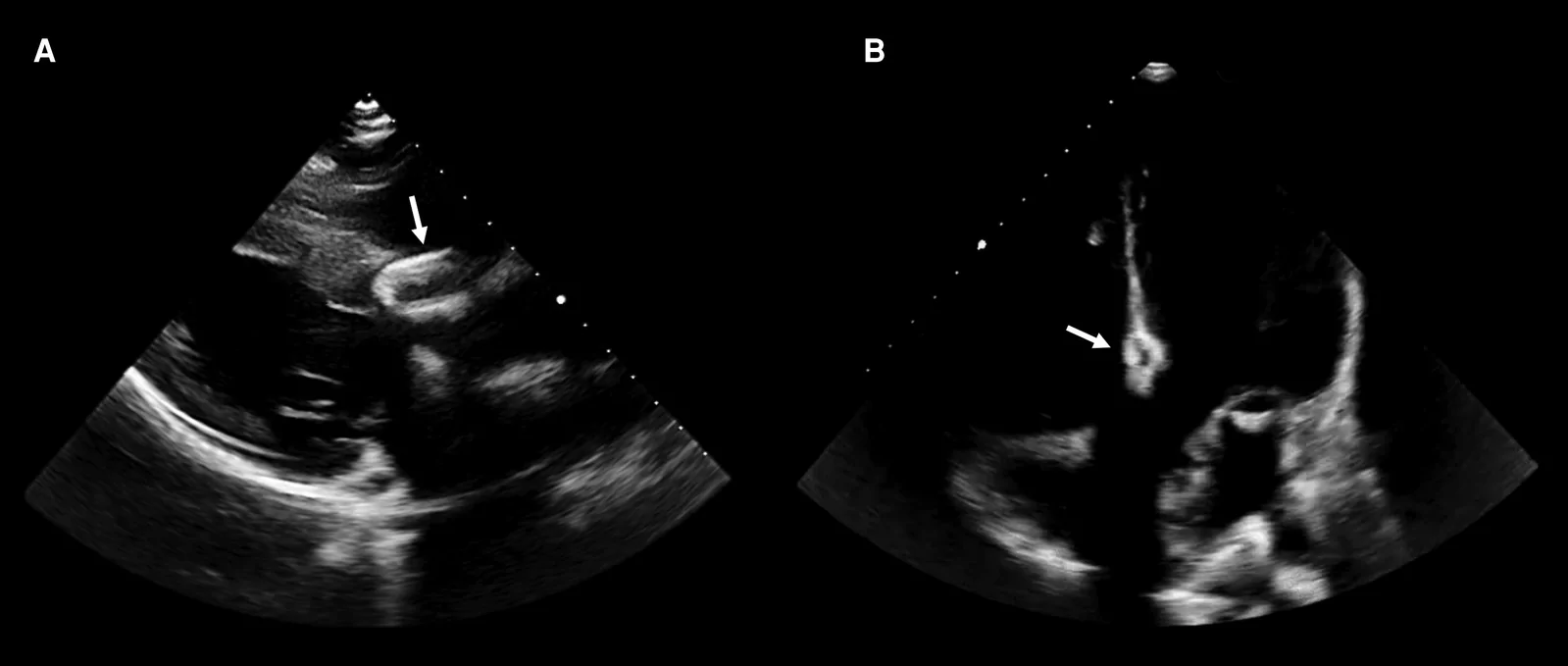
Transthoracic echocardiography. (*A*) Parasternal long-axis view and (*B*) apical four-chamber view, demonstrating a rounded lesion with hyperechoic border and anechoic interior in the basal anterior interventricular septum (arrows).

Cardiac magnetic resonance (CMR) imaging demonstrated a 20 mm well-circumscribed intramyocardial lesion in the basal anterior interventricular septum, isointense on T1-weighted sequences, and hyperintense on STIR, with peripheral gadolinium uptake on first-pass perfusion and peripheral late gadolinium enhancement (LGE), without evidence of myocardial fibrosis in the surrounding segments (*Figure 3*). Biventricular volumes were increased; left ventricular ejection fraction was preserved at 54%, while right ventricular ejection fraction was at the lower limit of normal at 40%.

**Figure 3:**
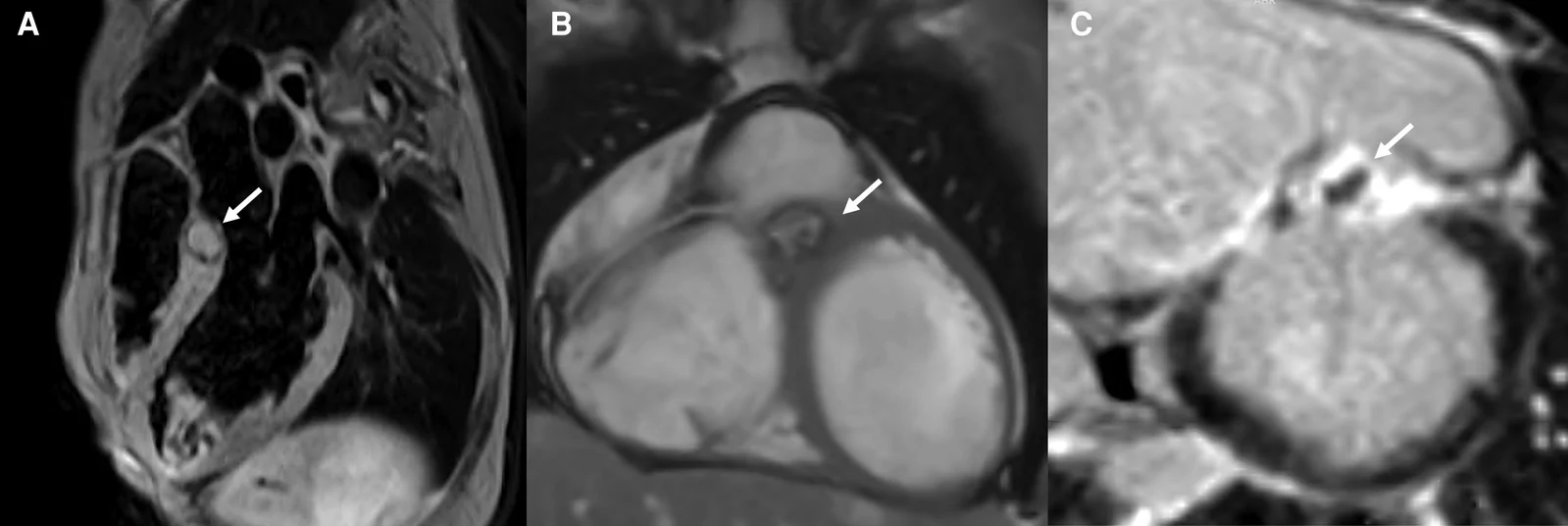
Cardiac magnetic resonance imaging. (*A*) T1 fat-saturated sequence, three-chamber view; (*B*) STIR sequence, four-chamber coronal view; and (*C*) late gadolinium enhancement, parasternal short-axis view. All sequences demonstrate an intramyocardial lesion in the basal anterior interventricular septum (arrows), isointense on T1-weighted imaging, with STIR hyperintensity consistent with fluid cystic content and peripheral late gadolinium enhancement consistent with a fibrous pericyst.

Whole-body computed tomography (CT) (*Figure 4*) identified a 3 × 1.6 cm lobulated annular calcification in the basal interventricular septum consistent with the calcified fibrous pericyst of a hydatid cyst and a second lesion of 1.7 × 1.2 cm in the AV pericardial recess indicating multifocal cardiac involvement. No extracardiac hydatid lesions were identified on CT or hepatic MRI. The larger dimensions on CT compared with CMR (20 mm) reflect the distinct components measured by each technique. Given the characteristic multimodal cardiac imaging findings and peripheral eosinophilia, probable hydatid disease due to *E. granulosus* was established as the leading diagnosis.

**Figure 4:**
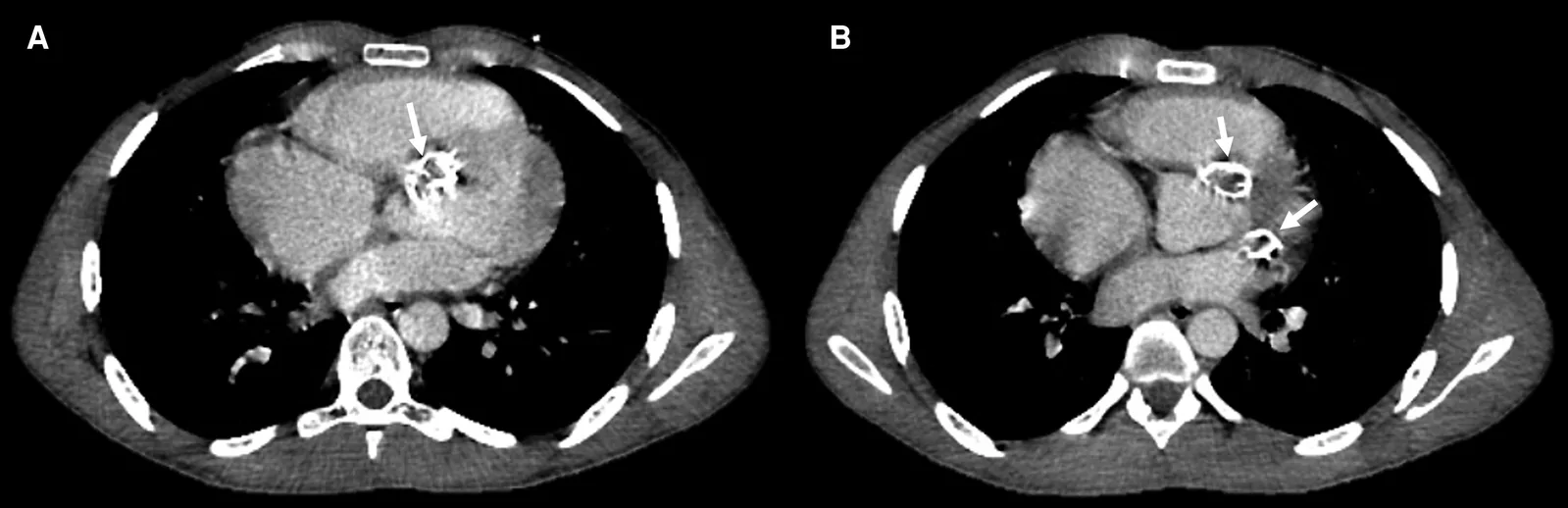
Computed tomography of the chest, axial view, soft tissue window, demonstrating a 3 × 1.6 cm lobulated annular calcification in the basal interventricular septum (*A*) and a second lesion of 1.7 × 1.2 cm in the atrioventricular pericardial recess (*B*).

Empirical albendazole (400 mg twice daily) was initiated during admission and continued for 6 months after discharge. Pacemaker implantation was deferred until the imaging work-up was completed and active disease was excluded; a permanent dual-chamber pacemaker was successfully implanted via left axillary access on Day 22 of admission. The patient was discharged in stable condition and remained clinically stable at 1-year follow-up. Device interrogation revealed 99% ventricular pacing, confirming pacemaker dependency. Peripheral eosinophilia had resolved on follow-up blood tests. A third *E. granulosus* serological test was also negative.

## Discussion

This case highlights the importance of multimodal imaging in the evaluation of unexplained conduction disease in young patients from endemic regions. The characteristic echocardiographic appearance, a rounded lesion with hyperechoic border and anechoic interior, should prompt a multimodal diagnostic work-up even without serological confirmation.^5^

Cardiac hydatidosis most frequently involves the left ventricle (55%–60%), with septal location in 4%–10% of cases.^2,4^ Multifocal cardiac involvement, as demonstrated here by the concomitant pericardial recess lesion, occurs in up to 10% and warrants comprehensive CT assessment of all cardiac structures.^2^ Septal location carries particular clinical significance: the basal anterior interventricular septum is the anatomical region through which the His bundle courses after the compact AV node, and direct involvement by the cyst at this level represents the most plausible mechanism of complete AV block in the present case.^2^

The diagnosis in this case fulfils criteria for probable rather than confirmed cardiac echinococcosis, given the absence of histopathological confirmation, negative *E. granulosus* serology, and the lack of molecular testing. However, imaging-based diagnosis is the accepted standard for intracardiac hydatid cysts, where pathological confirmation is rarely obtained without surgical intervention.^4,6^

Cardiac magnetic resonance added decisive diagnostic value: STIR hyperintensity reflecting fluid cystic content combined with peripheral LGE from the fibrous pericyst enables differentiation from tumours, abscesses, and other intramyocardial masses.^7^ The serological findings in this case deserve specific comment. While the sensitivity of *E. granulosus* serology is relatively high for hepatic cysts, serological tests are false-negative in up to 50% of cardiac cysts.^3^ In calcified cysts, as seen here on CT, sensitivity is further reduced, as calcification reflects chronic or involuting disease with minimal viable antigen.^3^ According to the World Health Organization classification of cystic echinococcosis, calcified cysts correspond to stage CE5, the inactive stage, in which viable protoscolices are unlikely, rendering serological tests practically uninformative; seronegativity in this context is therefore a coherent rather than a contradictory finding. However, it should be noted that this classification was developed primarily for hepatic cysts and has not been formally validated for cardiac lesions, though its conceptual application remains pathophysiologically justified.^4^

The differential diagnosis of an intramyocardial cystic lesion also includes entities such as cardiac fibroma with dystrophic calcification, tuberculoma, or calcified granulomas. In the present case, the combination of multimodal imaging findings (anechoic interior, STIR hyperintensity, and annular calcification) excludes solid or fibrous lesions and taken together supports probable hydatid disease as the primary diagnosis.^7^

Surgical resection remains the definitive treatment for large or high-risk cardiac hydatid lesions.^6^ In the present case, the decision was made by a multidisciplinary heart team. Conservative management was chosen based on the complex intraseptal surgical location^2,6^ and the calcified appearance of the lesions suggesting inactive disease. A watchful waiting strategy with antiparasitic therapy was therefore considered appropriate.

With increasing migration from endemic regions to Europe, probable cardiac hydatidosis must remain in the differential diagnosis of intracardiac masses and unexplained conduction abnormalities in young patients, even when serological confirmation is absent or misleading.

## Conclusion

We describe a case of probable cardiac hydatidosis presenting as complete AV block in a 16-year-old immigrant from Senegal, in which multimodal imaging strongly supported the diagnosis despite negative *E. granulosus* serology. This case illustrates that serological results, whether negative or misleadingly positive for other parasites, must be interpreted in the context of imaging findings and that a structured multimodal approach is indispensable for the diagnosis of cardiac parasitic masses in patients from endemic regions.

## Lead author biography


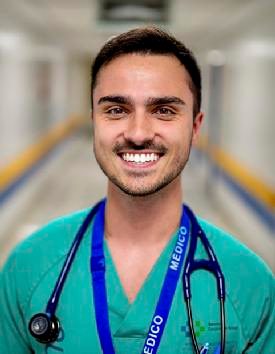


Diego López Fazlic, MD, is a fourth-year cardiology resident at Hospital Universitario Nuestra Señora de Candelaria, Santa Cruz de Tenerife, Spain. His clinical interests include cardiac imaging and clinical cardiology research. He is affiliated with the European Society of Cardiology (ESC) and is a member of the Spanish Society of Cardiology (SEC).

## Supplementary Material

ytag789_Supplementary_Data

## Data Availability

Data sharing is not applicable to this article as no datasets were generated or analysed. De-identified clinical images are available from the corresponding author upon reasonable request.
